# Supplementary material for: Hamiltonian modelling of macro-economic urban dynamics
Source: R Soc Open Sci. 2020 Sep 23;7(9):200667. doi: 10.1098/rsos.200667 (PMC7540774; doi:10.1098/rsos.200667)
Supplement: Supporting Information Pdf [file rsos200667supp1.pdf]

Hamiltonian Modeling of Macro-Economic Urban Dynamics: Supporting Information

Bernardo Monechi[1], Miguel Ibáñez-Berganza[2], and Vittorio Loreto[1,2,3]

[1] *Sony Computer Science Laboratories, 6, Rue Amyot, 75005, Paris, France*
[2] *Sapienza University of Rome, Physics Department, Piazzale Aldo Moro 2, 00185, Rome, Italy*
[3] *Complexity Science Hub Vienna, Josefstädter Strasse 39, A-1080 Vienna, Austria*

S1 INSEE Data about "Communes" in France

The data we consider in this work comes from the French Institut National de la Statistique et des Études Économiques (INSEE) and can be downloaded freely from its website (`https://www.insee.fr/fr/accueil`). The data we downloaded concerns several aspects of the French "Communes" which are the smallest administrative units in the country, ranging from few hundreds of inhabitants to several millions. In our analysis we arbitrarily removed all the administrative units with less than 20 inhabitants. There is much information in the data we downloaded that has not been used in the work, while other has been aggregated to obtain 10 socio-economic indicators representing some aspects of the job market and of the population. INSEE provides yearly snapshots of data about the communes. In our work, we downloaded data from 2006 to 2015, from different data sources. In the following we indicate with $\{Y\}$ a variable which is the last two digits of each year (e.g. $\{Y\} = 12$ in 2012).
From "Emploi - Population active" data we built the variables

1. **Jobs in Primary and Secondary Sectors**: the sum of the variables C$\{Y\}$_EMPLT_CS1 (agriculture operators), C$\{Y\}$_EMPLT_CS6 (factory workers), C$\{Y\}$_EMPLT_AGRI (workers in agriculture), C$\{Y\}$_EMPLT_INDUS (employed in industry), C$\{Y\}$_EMPLT_CONST (workers in construction).
2. **Jobs in the Tertiary Sector**: C$\{Y\}$_EMPLT_CS4 (intermediary professions)
3. **Jobs in Commerce**: the sum of C$\{Y\}$_EMPLT_CTS (workers in commerce) and C$\{Y\}$_EMPLT_CS2 (works in artisan's shops)
4. **Jobs in Quaternary**: C$\{Y\}$_EMPLT_CS3 (workers in intellectually superior jobs)
5. **Jobs in Public Administration and services**: C$\{Y\}$_EMPLT_APESAS (workers in public administration, teaching, health institutes and social aid).
6. **Employment rate**: ratio between P$\{Y\}$_ACTOCC1564 (active employed population) and P$\{Y\}$_ACT1564 (active population).z

From "Diplômes - Formation" data we built the variables:

7. **Fraction of highly educated**: ratio between P$\{Y\}$_NSCOL15P_SUP (Population not in school more than 15 years old with higher education degrees) and P$\{Y\}$_NSCOL15P (Population not in school more than 15 years old)

From "Population par sexe, âge et situation quant á l'immigration"

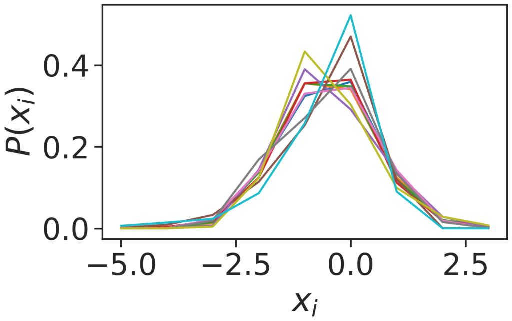

Figure S1: Distributions for the re-scaled x_i variables for the year 2012. The other years are not shown, but similar results can be found in those cases.

8. **Number of immigrants**: sum of AGE400_IMMI1_SEXE1, AGE400_IMMI1_SEXE2, AGE415_IMMI1_SEXE1, AGE415_IMMI1_SEXE2, AGE425_IMMI1_SEXE1, AGE425_IMMI1_SEXE2, AGE455_IMMI1_SEXE1, AGE455_IMMI1_SEXE2. Here SEXE1 indicates males and SEXE2 indicates females. Moreover, AGE400 indicates population less than 15 years old, AGE415 population of age between 15 and 24, AGE425 population of age between 24 and 54, AGE455 population more than 54 years old.

From "Salaires et revenus d'activité" data:

9. **Average salary per hour**: the variable SNHM$\{Y\}$.

Finally the population of each commune can be read from the "Evolution et structure de la population" data, in the P$\{Y\}$_POP variable. Each variable X_i has been found to be dependent on the population P via the power-law relation $X_i = X_i^0 P_i^a$. The exponents a_i for each variable in each year are shown in the main text and are found to be roughly constant in time. We use this relation to define the variable $x_i = \log_{10}(X_i/(X_i^0 P^{a_i}))$, which then we re-scale by their standard deviation $\sigma(x_i)$. In this way, we obtain variable which are bell-shaped with the same variance as shown in Fig. S1.

S2 Error Estimation with Bootstrapping and t-test

To perform the t-tests in the main text we need to estimate the error on our observables $C^{(n)}$. Considering a certain function $f(x)$ defined on our data $\{x^\alpha\}_{\alpha=1}^{N_c}$, we can easily estimate its average over the sample using

$$\langle f \rangle_{data} = \frac{1}{N_c} \sum_\alpha f(x^\alpha). \tag{1}$$

In order to assign an error to the average we can divide our sample in M sub-samples of $0.8N_c$ elements, built by randomly picking elements of $\{x^\alpha\}_{\alpha=1}^{N_c}$ (with repetitions). We can then use (1) on each sub-sample, finding a set of mean values $\{\langle f \rangle_m\}_{m=1}^{M}$ (bootstrap sample) where m identifies different sub-samples. The average over this set can the be assumed as an estimate of $\langle f \rangle_{data}$. Similarly, the standard deviation over the set $\{\langle f \rangle_m\}_{m=1}^{M}$ can be assumed as standard error. We indicate this two quantities with $\bar{f}$ and $\sigma(f)$ respectively. We can use the set $\{\langle f \rangle_m\}_{m=1}^{M}$ to perform a t-test to check the compatibility of $\langle f \rangle_{data}$ with a certain value μ_f, via the statistics

$$t_{1sample} = \frac{\bar{f} - \mu_f}{\sigma(f)}. \tag{2}$$

This statistics is used to perform a double-tailed test over the t-distribution with $M-1$ degrees of freedom under the null hypothesis that $\bar{f}$ is different from μ_f. We reject this hypothesis if the p-value of the test is larger than 0.05. In case we need to compare two empirical averages (e.g. when we compared the components of $C^{(4)}$ and those of $(C_{gauss})^{(4)}$), we build a bootstrap sample for each quantity. Identifying these quantities with f and g respectively and with M_f and M_g the dimension of the bootstrapped sample, we use the statistics

$$t_{2sample} = \frac{\bar{f} - \bar{f}}{\sqrt{\sigma(f)^2 + \sigma(g)^2}}. \tag{3}$$

to perform a double tailed test with a t distribution with

$$\nu = \frac{\sigma(f)^2 + \sigma(g)^2}{\frac{\sigma(f)^4}{M_f - 1} + \frac{\sigma(g)^4}{M_g - 1}}, \tag{4}$$

degrees of freedom under the null hypothesis that $\bar{f}$ and $\bar{g}$ are different. We can use this test also to compare empirical averages with those produced by the model exchanging the bootstrapped average and standard error with those obtained with a Langevin simulation (in which case the size of the sample is the number of simulation steps).

S3 Maximum Entropy and Parameters Estimation

Let's consider a data set of N_c points that can be considered several realizations of the same distribution $\{x^\alpha\}_{\alpha=1}^{N_c}$. Each $x^\alpha \in \mathcal{R}^N$ and we indicate with x_i^α its i-th component. Suppose we have identified a set of observables $O_\lambda(x)$ with λ an integer index, which are function of $\mathcal{R}^N$ and are relevant for the description of our dataset. Maximum Entropy (ME)[1] provides an interesting framework for deriving a generative model which preserves the average of the observables measured with the data, $\langle O_\lambda \rangle_{data}$. In ME the goal is to find a distribution $\mathcal{P}(x)$ maximising its entropy under the constraints that the average of the observables computed with $\mathcal{P}(x)$ should be the same as in the data. In other words, in ME we have to maximize the functional:

$$\Gamma[\mathcal{P}] = S[\mathcal{P}] + \sum_\lambda J_\lambda \left(\langle O_\lambda \rangle_{data} - \langle O_\lambda \rangle_{\mathcal{P}}\right), \tag{5}$$

where $S[\mathcal{P}] = -\int dx \mathcal{P}(x) \log \mathcal{P}(x)$ is the Entropy of the distribution $\mathcal{P}$ and $\langle f \rangle_{\mathcal{P}} = \int dx f(x) \mathcal{P}(x)$ is the average of the function f over the distribution $\mathcal{P}$. In other words, equation (5) is the Lagrangian function which maximises the entropy under the constraints that the observables produced by $\mathcal{P}$ should be the same as those in the data. Hence, J_λ are the Lagrange multipliers related to each constraint. With some straightforward calculations, we can show that maximizing equation (5) with respect to $\mathcal{P}$, is equivalent to maximize the loglikelihood

$$\mathcal{L}(J_\lambda) = \frac{1}{N_c} \sum_\alpha \log \mathcal{P}(x^\alpha; J_\lambda), \tag{6}$$

with respect to J_λ, where $\mathcal{P}$ is defined as

$$\mathcal{P}(x) = \frac{1}{Z} \exp\left(-\sum_\lambda J_\lambda O_\lambda(x)\right). \tag{7}$$

In equation (7) Z the "partition function" well-known in Statistical Physics and $H(x) = \sum_\lambda J_\lambda O_\lambda(x)$ is the Hamiltonian function of the system. It is possible to show that maximizing equation (6) equals to solve the equations:

$$\frac{\partial \mathcal{L}}{\partial J_\lambda} = \langle O_\lambda \rangle_{\mathcal{P}} - \langle O_\lambda \rangle_{data} = 0. \tag{8}$$

However, this would require to know a closed form for $\langle O_\lambda \rangle_{\mathcal{P}}$ which is typically not the case. Another common approach to estimate the maximum of the likelihood function is that to perform a gradient ascent using equations (8). The problem with $\langle O_\lambda \rangle_{\mathcal{P}}$ at each step of the ascent is solved by using Langevin simulations to compute these averages and then use that to compute the gradients. This is the approach we have used for our system. Note that typically this is not feasible if the phase space becomes too big. However, in our case $N = 9$ allows to have estimates of $\langle O_\lambda \rangle_{\mathcal{P}}$ with reasonably short simulations.

S4 Maximum Entropy for rescaled socio-economic indicators

Considering the data in the main text, we are interested in estimating the effective interactions between the re-scaled indicators x_i. In the main text we have identified some observables related to the correlations between the indicators. In particular, we have seen that besides $C^{(2)}_{i,j} = \langle x_i x_j \rangle_{data}$ also $C^{(3)}_{i,j,k} = \langle x_i x_j x_k \rangle_{data}$ cannot be considered equal to 0. If we assume $C^{(2)}_{i,j}$ as the only relevant observables, according to the framework defined in the previous paragraph we would end up with a model

$$\mathcal{P}_0(x) \propto \exp\left(-\sum_{ij} J^{(2)}_{ij} x_i x_j\right), \tag{9}$$

i.e. a Gaussian model which is not capable of producing correlations $C^{(n)}$ with odd n. The fact that $C^{(3)}_{i,j,k}$ cannot be considered 0 forces us to assume it as a relevant observable to be put in the model. The inclusion of $C^{(3)}$ might lead to $C^{(1)}$ different from 0 which is instead observed in the data. Thus, we will include $C^{(1)}_i = 0$ for every i as an observable in the model. We obtain the model defined in the main text in which there is a contribution to the Hamiltonian of 3-points interactions

$$\mathcal{P}(x) \propto \exp\left(-\sum_{ij} J^{(2)}_{ij} x_i x_j - \sum_{ijk} J^{(3)}_{ijk} x_i x_j x_k + \sum_i J^{(1)}_i x_i\right). \tag{10}$$

The introduction of the term $J^{(3)}$ in the Hamiltonian make so that the distribution $\mathcal{P}(x)$ cannot be normalized if its domain is $\mathcal{R}^N$. In other words, there will be directions in $\mathcal{R}^N$ that will make the distribution grow indefinitely. However, there might be values of the coupling parameters that will allow for some local maxima that will constrain the dynamics of the system for a finite time, before it diverges for $t \to \infty$. To prevent this behaviour from happening, we can bound the system around these maxima redefining the domain of the probability $\mathcal{P}(x)$ so that $\mathcal{P} : I \to \mathcal{R}$ with $I = [-L, L]^N$ is a hypercube centred on the origin. The choice of the value L influences the final behaviour of the model, as well as on the training phase. If L is too small, some parts of the space that are populated by the empirical data could be excluded. A value which is too large might instead allow some of the diverging directions to appear within the hypercube. At each training step, we will generate a sample from the model by iterating the discrete Langevin equation (18) to have an approximation of the gradient of the likelihood function. Hence, these directions will make the dynamics collapse to the border of the hypercube, leading to an incorrect estimation. Fig. S4 shows the percentage of data points within the hypercube as a function of L. We see that the first value that almost all the sample if $L > 5$, hence we choose $L = 6$, i.e. 6 standard deviations of the sample.

To estimate the Lagrange multipliers $J^{(2)}_{ij}$ and $J^{(3)}_{ijk}$, we need to find the values maximizing (7) via gradient ascent. This requires to be able to compute exactly the log-likelihood Z to be computed. Estimating Z is quite a hard task typically. To circumvent this problem, we will use an approach widely used for training Energy Based models in Machine Learning[2, 3]. We can write the log-likelihood of our model as:

$$\mathcal{L} = -\langle H(x) \rangle_{data} - \log Z, \tag{11}$$

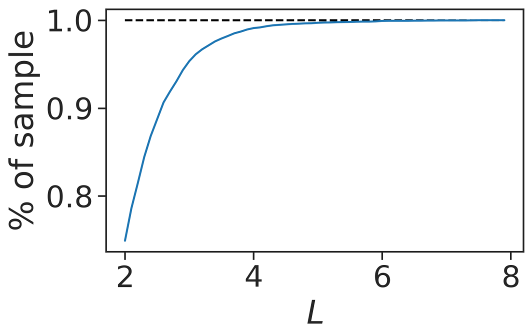

Figure S2: Percentage of data points contained withing the hypercube $I = [-L, L]^N$ as a function of L.

Where $\langle \cdot \rangle_{data}$ indicates the average on the sample data. Taking the gradient of the above expression we find

$$\nabla \mathcal{L} = -\langle \nabla H(x) \rangle_{data} + \langle \nabla H(x) \rangle_{\mathcal{P}}, \tag{12}$$

where $\langle \cdot \rangle_{\mathcal{P}}$ is the average for the model. This average can be approximated at each training step by averaging over a sample obtained with numerical simulations (e.g. by iterating equation (18). If we use this approximation we can see from equation (12) that maximising the log-likelihood is equivalent to optimise the cost function:

$$\mathcal{C} = -\langle H(x) \rangle_{data} + \langle H(x) \rangle_{\mathcal{P}}, \tag{13}$$

that we can use to monitor the development of the training.

To avoid over-fitting when estimating the parameters of the model, we divided the sample in a training set ($\approx 70\%$ of the whole sample) and test set (the remaining part). In order to make the two samples as similar as possible, we initially divided the whole sample in percentiles of the population distribution: from the 0th percentile to the 5th; from the 5th percentile to the 10th and so one. We divided each classes in training and test with the proportion of 70% and 30%, having in this way a global train and test sample with the same population distribution. This was done in order to not over-represent small cities which are more numerous that the large ones.
The algorithm used to estimate $J^{(2)}_{ij}$ and $J^{(3)}_{ijk}$ is then:

1. Starting at $t = 0$, we set $J^{(2)}_{ij} = 1/2\delta_{ij}$, $J^{(3)}_{ijk} = 0$ and $J^{(1)}_{i} = 0$, which is equivalent to a system of non-interactive variables with variance equal to 1. We estimate the starting value of the cost function (13) for the training and test data.

2. At each time step, we generate a sample from the current version of $\mathcal{P}(x)$, iterating equation (18) with $dt = 0.1$ for at least 10^6 steps. To prevent the simulations from diverging, we bound the dynamics to the box $I = [-6, 6]^N$.

3. We use the generated sample and the training data to estimate the gradients

$$\begin{aligned} \frac{\partial \mathcal{L}}{\partial J^{(1)}_{i}} &= \langle x_i \rangle_{data} - \langle x_i \rangle_{\mathcal{P}}, \\ \frac{\partial \mathcal{L}}{\partial J^{(2)}_{ij}} &= \langle x_i x_j \rangle_{\mathcal{P}} - \langle x_i x_j \rangle_{data}, \\ \frac{\partial \mathcal{L}}{\partial J^{(3)}_{ijk}} &= \langle x_i x_j x_k \rangle_{\mathcal{P}} - \langle x_i x_j x_k \rangle_{data} \end{aligned} \tag{14}$$

4. We update the parameters using

$$\begin{aligned} J_i^{(1)} &\leftarrow J_i^{(1)} + \eta_J^{(1)} \frac{\partial \mathcal{L}}{\partial J_i^{(1)}}, \\ J_{ij}^{(2)} &\leftarrow J_{ij}^{(2)} + \eta_J^{(2)} \frac{\partial \mathcal{L}}{\partial J_{ij}^{(2)}}, \\ J_{ijk}^{(3)} &\leftarrow J_{ijk}^{(3)} + \eta_J^{(3)} \frac{\partial \mathcal{L}}{\partial J_{ijk}^{(3)}} \end{aligned} \tag{15}$$

5. We compute the new value of the cost function (13) for the training and test data and we update t by 1.

6. We restart from point 2 until the test log-likelihood stops growing.

The perturbative form for Z used to estimate the log-likelihood requires the contribution of $J^{(3)}$ to be smaller than that of $J^{(2)}$. Hence, we set $\eta_J^{(2)} = 0.01$ and $\eta_J^{(1)} = \eta_J^{(3)} = 0.001$. In Fig. S3 we show the cost function curves for all the train and test data for some years. We can see that we reach convergence quite quickly. The curves for the training and test sets are very similar. We can conclude that there are not overfitting issues and the model would generalize to non-observed data.

We can study the effects of $J^{(3)}$ in the Hamiltonian and the finite domain I to the distribution of the models. Considering the Gaussian model in equation (9), we can transform the space of the variables using projecting on the eigenvectors of the $J^{(2)}$ matrix. If we do so, the model becomes a set of non-interacting Gaussian models. Fig. S4 shows the comparison between the various distributions on the Principal Components (PC), i.e. the directions of the eigenvalues of $J^{(2)}$, between the model and the data. We can see that on each PC the model (9) predicts a Gaussian distribution centred in 0 (orange line) which is not so far from the empirical distribution (blue bars). Doing the same for the model in equation (10) gives a slightly different result. We can see in Fig. S4 that the introduction of a bounded dominion allows the model to reproduce the bell-shaped distribution we see from the data (green line). However, the introduction of $J^{(3)}$ modifies the shape distribution tails, which are now more adherent to the empirical one.

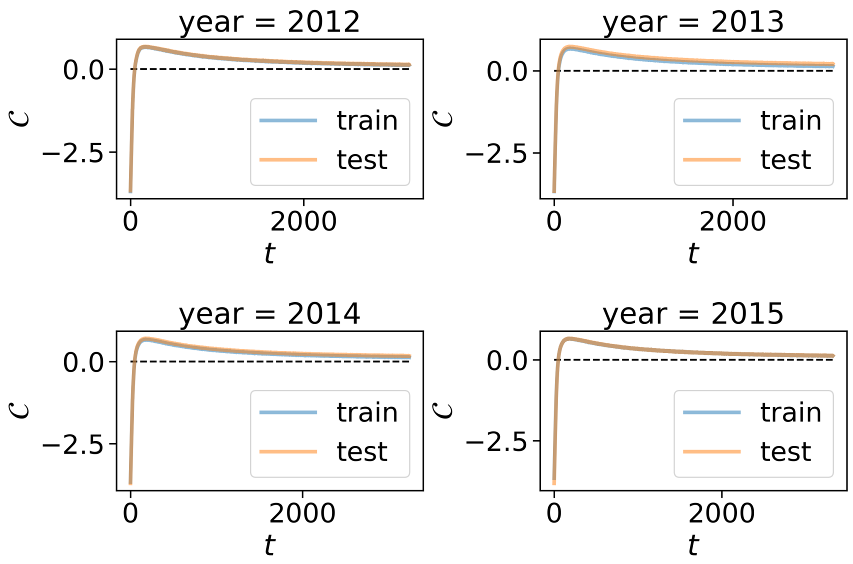

Figure S3: Cost function for the train and test data during training as a function of the training step.

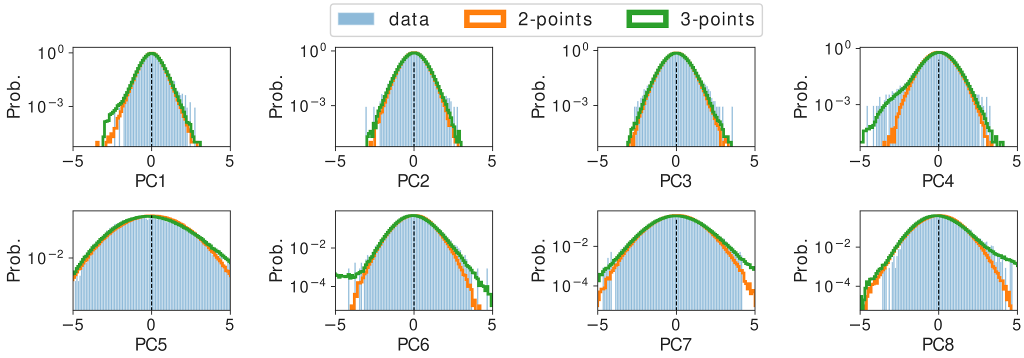

Figure S4: Ditribution of the Pricipal Components of $J^{(2)}$ obtained with the data (blue bars), the Gaussian model of equation (9) (orange line) and with the model with 3-points interactions and bounded dominion (10) (green line).

S5 Other Examples of Prediction of a Dependent Variable

In the main text we have shown some examples of predictions of the model, when an indicator is chosen as a dependent variable and another two are used as the independent ones. In Fig. S5 , we show some other examples for the $\mathcal{P}_0$ (only $C^{(2)}$ correlations are use in the model) and the $\mathcal{P}$ (also $C^{(1)}$ and $C^{(3)}$). We can see that the pattern observed in the main text is reproduced for almost every one of the shown cases, i.e. there is more agreement between of the model and the data if $J^{(3)}$ interactions are taken into account. Considering the fist and second columns of panels, the theoretical predictions are obtained as the average of the distributions $\mathcal{P}_0(x_i|x_j, x_k)$ and $\mathcal{P}(x_i|x_j, x_k)$, where x_i is the chosen dependent variable and x_j and x_k are the two chosen independent ones. Sampling from these distributions can be made by means simulating the corresponding Langevin dynamics (18),

keeping fixed the variables x_j and x_k at each step. In a similar, but more simple fashion we can study the dependence of just one indicator with respect to another. In Fig. S6 , we show four indicators - the employment rate, the fraction of highly educated people, the number the average salary per hour - as a function of the the number of jobs in the quaternary sector. It is evident that the introduction of the nonlinear term $J^{(3)}$ increases the model predictive ability, and it is key to capture the non-linear effects present in the data also in this more simple case. Let us take for instance panels (b) and (f) of Fig. S6 reporting the behaviour of the rescaled indicator for the fraction of highly educated citizens as a function of the rescaled indicator for number of jobs in the Quaternary sector . Panel (b) is reporting the comparison of the prediction of the full model with non-linear terms, with the empirical data. Panel (f) shows the same comparison for a simpler Gaussian model described without $J^{(3)}$. We can see that in this case, when the rescaled indicators for jobs in the Quaternary sector is smaller than 1, the increase in the Fraction of Highly Educated citizens is relatively small. In this region, this indicator is always close to 0, indicating a commune with an average number of highly educated individuals. For values above 1 (i.e., the number of this jobs in the Quaternary sector is more than 1 standard deviation to the average of the communes with the same population), the rescaled fraction of highly educated citizens starts to increase more rapidly. The model with only binary interactions fails to predict this behaviour, which is instead well reproduced by the model in with higher-order interactions. This result stays valid for other rescaled indicators as the employment rate (panels (a) and (e) of Fig. S6) and the average yearly salary (panels (d) and (h) of Fig. S6). In the case of the number of immigrants (panels (g) and (c)), no non-linear behaviour is present in the data, and both the models predict the dependence correctly on the rescaled values of the number of jobs in the Quaternary sector.

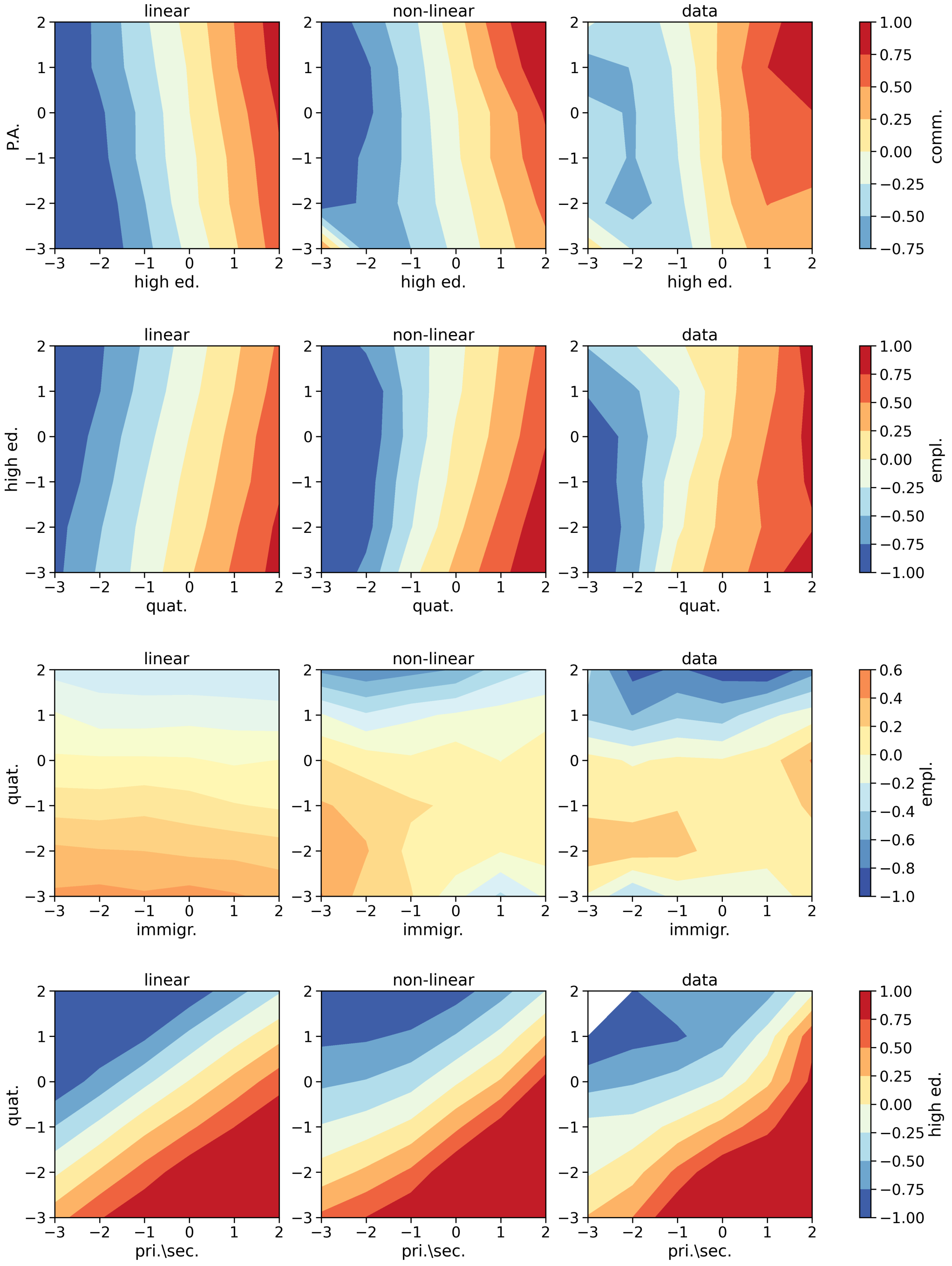

Figure S5: Some rescaled indicators (indicated in the label of the colorbar) as functions of the some couples of rescaled indicators (indicated on the x-axis and the y-axis of each panel). Areas in red (blue)represents communes with a large(small) value of the rescaled indicator used as dependent variable. The first column are the results obtained with the Hamiltonian model without the terms $J^{(1)}$ and $J^{(3)}$. The second column are the results obtained with the complete model including those terms. The last column of panels are the results obtained by binning the points for the communes in the year (2012).

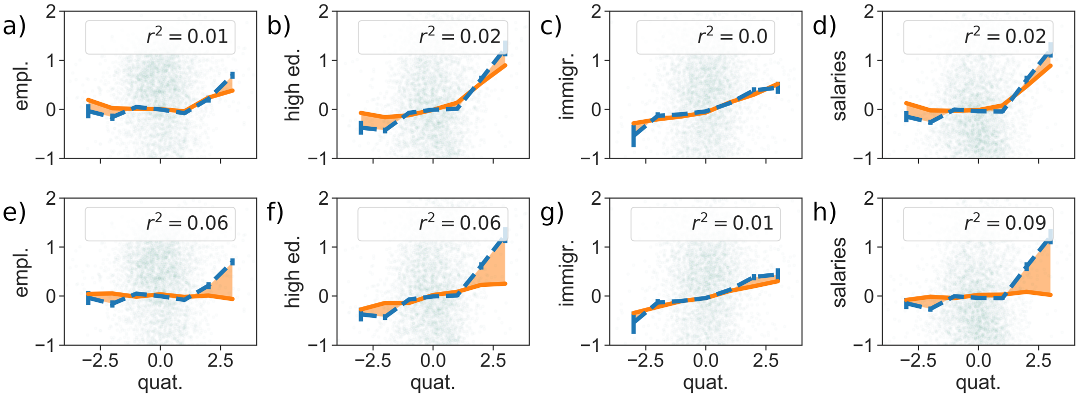

Figure S6: Rescaled indicators for *Employment rate*, *Fraction of highly educated people*, *Number of immigrants*, *Average salary per hour* indicators as a function of the rescaled indicator for number of jobs in the quaternary sector as measured with the empirical data (dashed blue line) and with two different Hamiltonian model, the complete one (upper row of panels) of model in the maix text and the simple Gaussian one (lower row of panels) without the terms $J^{(1)}$ and $J^{(3)}$ (orange lines) . Green points represent all the points in the data-set for the considered year (2012). The mean squared errors between the data and the models are also shown.

S6 Stationarity of the Inferred Models

Indicating with $J^{(2)}(y_1)$ and $J^{(3)}(y_1)$ the parameters inferred for the data in a certain year y_1, it is possible to compare them with those of another year y_2. To make statistical comparisons, it is needed to have an idea of the errors associated with each inferred parameter. Errors for the parameters can be computed using the Fisher Information matrix $\mathcal{I}$. In fact, the parameters estimated with Maximum Likelihood can be considered as a coming from a multivariate normal distribution, whose averages are the real parameters and the co-variance matrix is given by the inverse of $\mathcal{I}$. For our system $\mathcal{I}$ is defined as:

$$
\begin{aligned}
\mathcal{I}(J^{(2)}_{ij}, J^{(2)}_{lm}) &= -\frac{\partial}{\partial J^{(2)}_{ij}} \frac{\partial}{\partial J^{(2)}_{lm}} \log Z = C^{(4)}_{ijlm} - C^{(2)}_{ij} C^{(2)}_{lm} \\
\mathcal{I}(J^{(2)}_{ij}, J^{(1)}_{k}) &= -\frac{\partial}{\partial J^{(2)}_{ij}} \frac{\partial}{\partial J^{(1)}_{k}} \log Z = C^{(3)}_{ijk} - C^{(2)}_{ij} C^{(1)}_{k} \\
\mathcal{I}(J^{(2)}_{ij}, J^{(3)}_{lmn}) &= -\frac{\partial}{\partial J^{(2)}_{ij}} \frac{\partial}{\partial J^{(3)}_{lmn}} \log Z = C^{(5)}_{ijlmn} - C^{(2)}_{ij} C^{(3)}_{lmn} \\
\mathcal{I}(J^{(3)}_{ijk}, J^{(3)}_{lmn}) &= -\frac{\partial}{\partial J^{(3)}_{ijk}} \frac{\partial}{\partial J^{(3)}_{lmn}} \log Z = C^{(6)}_{ijklmn} - C^{(3)}_{ijk} C^{(3)}_{lmn}. \\
\mathcal{I}(J^{(3)}_{ijk}, J^{(1)}_{l}) &= -\frac{\partial}{\partial J^{(3)}_{ijk}} \frac{\partial}{\partial J^{(1)}_{l}} \log Z = C^{(4)}_{ijkl} - C^{(3)}_{ijk} C^{(1)}_{l} \\
\mathcal{I}(J^{(1)}_{i}, J^{(1)}_{l}) &= -\frac{\partial}{\partial J^{(1)}_{i}} \frac{\partial}{\partial J^{(1)}_{l}} \log Z = C^{(2)}_{il} - C^{(1)}_{i} C^{(1)}_{l}
\end{aligned}
\tag{16}
$$

To compute $\mathcal{I}$ we generate a sample from $P(x) \propto \exp(-H(x))$ iterating equation (18) with $dt = 0.1$ for at least 10^6 steps. We then use the produced sample to estimate the observables $C^{(n)}$. The errors associated to each parameter will be then computed using the corresponding element on the diagonal of $\mathcal{I}^{-1}$ as variance, and in turn using such variance to compute the standard error. As an example,

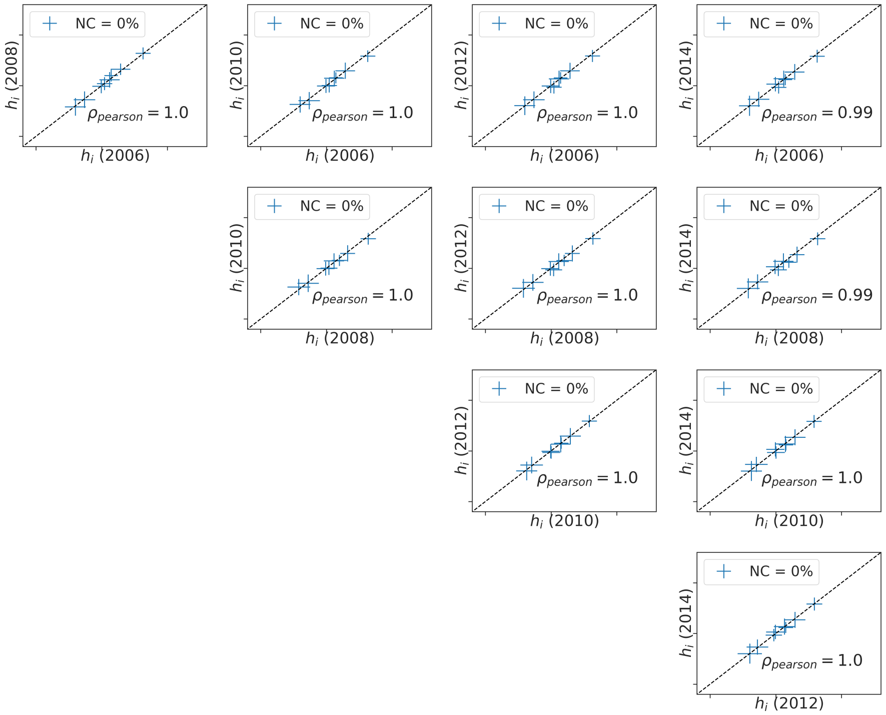

Figure S7: Comparisons between the components of $J^{(1)}$ in different years. Errors of each parameter are reported in the plot. Dotted line represents the identity relation. The percentage of component with a p-value from t-test below 0.05 is shown in the legend.

the standard error of the estimate of $J_{ij}^{(2)}$ is given by $\sqrt(\mathcal{I}^{-1}(J_{ij}^{(2)}, J_{ij}^{(2)})/N_c)$, where N_c is the number of points in the training set. Once we have computed all the errors for each components of $J^{(1)}(y)$, $J^{(2)}(y)$ and $J^{(3)}(y)$ for each year, we can make t-tests for each one of their components with null hypothesis that they are compatible. We reject the null hypothesis if the p-value of the test is larger than 0.05. Fig. S8 and Fig. S9 show the scatter-plot of the corresponding components of $J^{(1)}$, $J^{(2)}$ and $J^{(3)}$ for different years. Each component is plotted with its error and the percentage of components that have failed the t-test are shown in the legend of each plot. We can see from these figures that the parameters are quite similar between different years and typically the hypothesis of compatibility cannot be rejected.

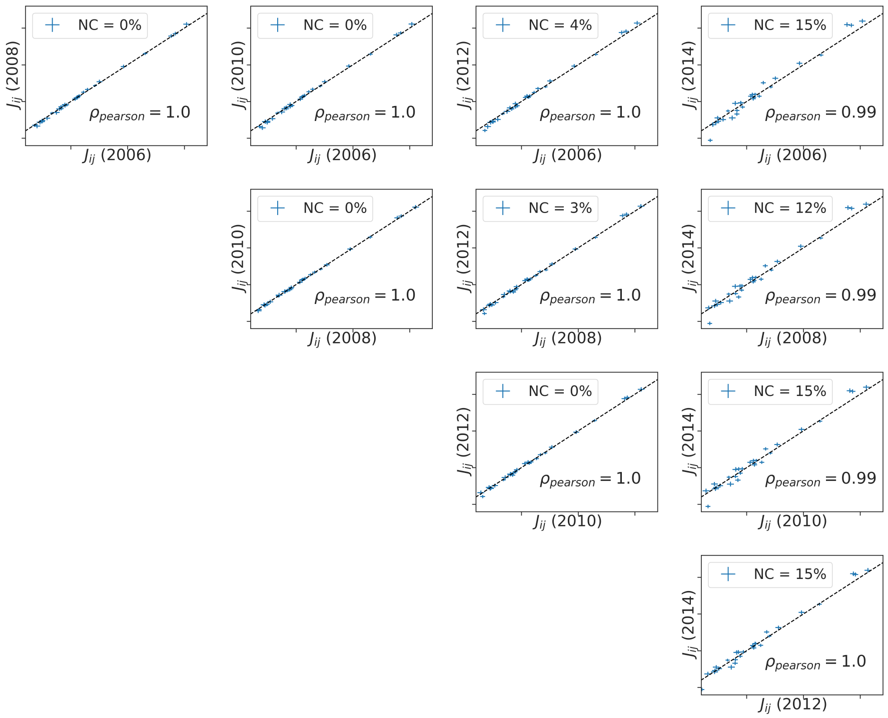

Figure S8: Comparisons between the components of $J^{(2)}$ in different years. Errors of each parameter are reported in the plot. Dotted line represents the identity relation. The percentage of component with a p-value from t-test below 0.05 is shown in the legend.

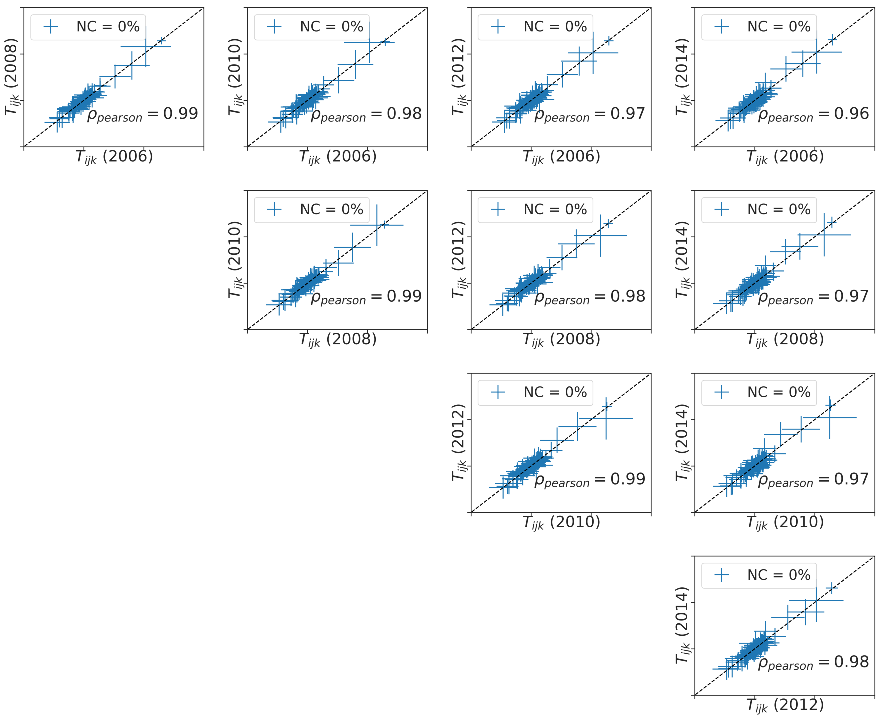

Figure S9: Comparisons between the components of $J^{(3)}$ in different years. Errors of each parameter are reported in the plot. Dotted line represents the identity relation. The percentage of component with a p-value from t-test below 0.05 is shown in the legend.

S7 Maximum Likelihood Estimation of the dt parameter

Assuming that our system can be described by a Langevin equation in the form of

$$\frac{dx}{dt}(t) = -\nabla H(x) + \eta(t), \tag{17}$$

it is easy to derive a discrete version of this equation, capable of coping with the discrete nature of the data we have. Supposing to have a small shift in time dt and calling $t' = t + dt$ we have

$$x(t') = x(t) - \nabla H(x(t))dt/2 + \eta\sqrt{dt}. \tag{18}$$

We assumed in the main text that the each component of the noise η_i is distributed according to a Laplace distribution with variance 1 and that different components are uncorrelated. Hence, each component of the vector $(x(t') - x(t) + \nabla H(x(t))dt)/\sqrt{dt}$ will be a Laplace-distributed variable. This simple fact allows to compute the transition probability from $x(t)$ to $x(t')$, that will be in the form

$$\mathcal{P}_{dt}(x(t')|x(t)) = \prod_{j=1}^{N} \sqrt{\frac{1}{2dt}} \exp\left(-\frac{\sqrt{2}|x_j(t+dt) - x_j(t) - (\partial H/\partial x_j)(\vec{x}(t))dt/2|}{\sqrt{dt}}\right). \tag{19}$$

At this point we would like to match the intrinsic time t of the model, with the real time of the data. To do so, we need to understand which dt corresponds to a time frame of one year. We can use Maximum Likelihood to fix this value, trying to maximize the Log-likeihood obtained by applying (19). In other words, we look for the value of dt maximizing the probability of observing the transitions we have in the data. Such log-likelihood can be written as

$$\mathcal{L}(dt) = \frac{1}{4N_C} \sum_{\alpha} \sum_{y=2006}^{2015} \mathcal{P}_{dt}(x^\alpha(t_{y+1})|x^\alpha(t_y)), \tag{20}$$

where $x^\alpha(t_y)$ is the vector of indicators of the city α in the year y (the notation t_y indicates the intrinsic time corresponding to the year y). Fig. show log-likelihood as a function of dt. The maximum observed value of $\mathcal{L}$ has been found for $dt_{max} = 0.014$.

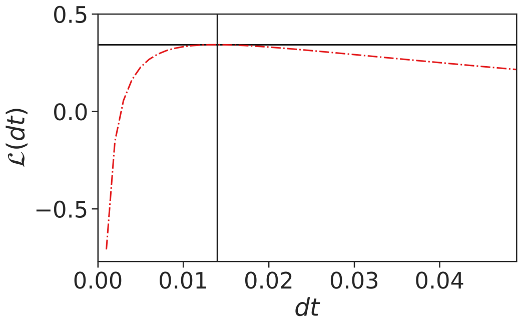

Figure S10: Log-likelihood in equation (20) as a function of dt. The maximum observed value of $\mathcal{L}$ is highlighted in the plot and has been found at $dt_{max} = 0.014$.

S8 Comparison with Causal Inference

The static model inferred in the main text is capable of predicting the evolution of a city if we use its corresponding Langevin equation to define a dynamics. In this case, we use the temporal information in the data only to infer the parameter dt used to make the Langevin equation discrete. Another approach we can use to define dynamic models is to use temporal correlations explicitly according to the Maximum Caliber principle[4]. First, we need to define time-dependent observables, i.e. observables depending on variable at different times. For sake of simplicity, we will focus on correlations of order 2 defined as,

$$C^{(2)}_{i,j}(\delta) = \langle x_i(y+\delta)x_j(y)\rangle_{data}, \tag{21}$$

where now the average is taken over all the communes in the data-set and all the years. As observables for the definition of the model we choose $C^{(1)}_i$, $C^{(2)}_{i,j}(\delta = 1)$. In this way, we are modelingng explicitly the average of the sample and the correlations between the indicators in consecutive years. The model corresponding to this set of observables has a transition probability defined by

$$\begin{aligned} &\mathcal{P}(x(y+1)|x(y)) \propto \\ &\exp\left(-\sum_i \frac{x_i(y+1)^2}{2} - \sum_{ij} B_{ij}x_i(y+1)x_j(y) + \sum_i c_i x_i(y+1)\right). \end{aligned} \tag{22}$$

This model corresponds to a linear model defined as

$$x(y+1) = -Bx(y) + h + \eta \tag{23}$$

where η is a normally distributed random variable with mean equal to 0 and variance equal to 1. Being equation (23) corresponding to a linear model, its parameters can be inferred by a standard linear regression.

References

[1] Martyushev LM, Seleznev VD. 2006 Maximum entropy production principle in physics, chemistry and biology. *Physics reports* **426**, 1–45.

[2] Du Y, Mordatch I. 2019 Implicit generation and generalization in energy-based models. *arXiv preprint arXiv:1903.08689.*

[3] LeCun Y, Chopra S, Hadsell R, Ranzato M, Huang F. 2006 A tutorial on energy-based learning. *Predicting structured data* **1**.

[4] Pressé S, Ghosh K, Lee J, Dill KA. 2013 Principles of maximum entropy and maximum caliber in statistical physics. *Reviews of Modern Physics* **85**, 1115.
